# Cascade screening in HBOC and Lynch syndrome: guidelines and procedures in a UK centre

**DOI:** 10.1007/s10689-024-00360-9

**Published:** 2024-03-13

**Authors:** D. Gareth Evans, Kate Green, George J. Burghel, Claire Forde, Fiona Lalloo, Helene Schlecht, Emma R. Woodward

**Affiliations:** 1 Manchester Centre for Genomic Medicine and North-West Genomics Hub, Manchester University Hospitals NHS Foundation Trust, Manchester, M13 9WL UK; 2 Division of Evolution Infection and Genomic Sciences, Faculty of Biology, Medicine and Health, Manchester Academic Health Science Centre, School of Biological Sciences, University of Manchester, Manchester, M13 9PL UK

**Keywords:** Cancer predisposition gene (CPG), Lynch syndrome, BRCA1/2, Genetic testing

## Abstract

In the 33 years since the first diagnostic cancer predisposition gene (CPG) tests in the Manchester Centre for Genomic Medicine, there has been substantial changes in the identification of index cases and cascade testing for at-risk family members. National guidelines in England and Wales are usually determined from the National Institute of healthcare Evidence and these have impacted on the thresholds for testing *BRCA1/2* in Hereditary Breast Ovarian Cancer (HBOC) and in determining that all cases of colorectal and endometrial cancer should undergo screening for Lynch syndrome. Gaps for testing other CPGs relevant to HBOC have been filled by the UK Cancer Genetics Group and CanGene-CanVar project (web ref. https://www.cangene-canvaruk.org/). We present time trends (1990–2020) of identification of index cases with germline CPG variants and numbers of subsequent cascade tests, for *BRCA1*, *BRCA2*, and the Lynch genes (*MLH1*, *MSH2*, *MSH6* and *PMS2*)*.* For *BRCA1/2* there was a definite increase in the proportion of index cases with ovarian cancer only and pre-symptomatic index tests both doubling from 16 to 32% and 3.2 to > 8% respectively. A mean of 1.73–1.74 additional family tests were generated for each *BRCA1/2* index case within 2 years. Overall close to one positive cascade test was generated per index case resulting in > 1000 risk reducing surgery operations. In Lynch syndrome slightly more cascade tests were performed in the first two years potentially reflecting the increased actionability in males with 42.2% of pre-symptomatic tests in males compared to 25.8% in *BRCA1/2* (*p* < 0.0001).

**Supplementary Information:**

The online version contains supplementary material available at 10.1007/s10689-024-00360-9.

## Introduction

The first Cancer Predisposition Gene (CPG) testing occurred in Manchester in 1990, with the discovery of the *TP53* gene as a high-risk gene for Li Fraumeni syndrome [1], followed by *BRCA1*, *BRCA2* and Lynch (*MLH1*, *MSH2*, *MSH6* and *PMS2*) genes in 1991–1995. This enabled the introduction of CPG testing into the cancer genetics clinics and allowed cascade testing to unaffected family members.

The detection of high-risk germline CPG variants in index cases makes available not only cancer prevention and treatment opportunities for that person but also, through family cascade testing, subsequent cancer prevention and early detection possibilities for at-risk family members who test positive for the CpG variant. Family members who test negative can, nearly always, be released from high-risk screening programmes, relieving stress and preventing overdiagnosis and false positive screening tests.

Prior to 2003 practice in cancer genetics in the UK was largely guided by the UK family cancer Study Group (later UK Cancer Genetics Group-CGG) for breast cancer [2] with those for bowel cancer largely coming from collaborations between geneticists and colorectal surgeons [3]. These early guidelines did not set specific thresholds for genetic testing but, emphasised the importance of testing high risk individuals with cancer to open the possibility of testing for *BRCA1, BRCA2* and Lynch syndrome genes [2, 3]. Indeed, the main thrust was identifying people at sufficient risk for early detection strategies.

No specific threshold was introduced for testing for HBOC until the 2004 NICE guideline [4] which set a 20% threshold for likelihood of *BRCA1/2* and only recommending testing in people affected with a relevant cancer to identify index cases for testing relatives. This later fell to a 10% threshold in 2013 [4] with allowance for testing unaffected relatives where no living affected was available for testing. Since then, NICE recommended testing all triple negative breast cancers aged < 50 years in a short update. More recently (2019-) testing has been governed by algorithms from NHS England’s Genomic Testing directory for rare and inherited disease, with the latest version at time of writing available online as version 5.2 in June 2023 (Table 1) [5]. This reflects testing criteria for all English centres. A Manchester score of ≥ 15 and the CanRisk model reflect a 10% threshold although some of the criteria allow testing at much lower levels. The latter recommendations include testing all non-mucinous epithelial ovarian cancer and male breast cancer cases, with testing of all triple negative breast cancer < 60years and all other breast cancer including high grade DCIS aged < 40years (Table 1). The testing directory reflects the switch in testing of index affected cases from medical genetics departments to mainstream testing in oncology or surgery. Mainstreaming for ovarian cancer in Manchester started in November 2017 [6] and for breast cancer in April 2021 [7]. The initial panel R208 only included *BRCA1, BRCA2* and *PALB2 with CHEK2* and *ATM* added in April 2022 and *RAD51C* and *RAD51D* in August 2022. Currently missense variants are not reported except the high penetrance c.7271T > G in *ATM*. Traditionally *CHEK2* and *ATM* were not considered ‘actionable’ genes in the UK and although *RAD51C* and *RAD51D* were considered actionable mainly from the ovarian risk point of view [8], this meant that cascade testing has been recommended [8]. There is still no clear guidance on cascade testing for *CHEK2* and *ATM* despite *CHEK2* c.1100delC being identified on clinical testing of *BRCA1 and BRCA2* as part of Copy Number Variant testing for *BRCA2* since around 2001 [9]. However, the recent inclusion of breast cancer risks estimation for both *CHEK2* and *ATM* in the CanRisk model [10] has led to offering testing beyond the initial proband in the last year.

**Table 1:** NHS England mainstream testing criteria since June 2023 for breast and other cancers for R208 (gene panel of *BRCA1, BRCA2, PALB2, CHEK2, ATM, RAD51C., RAD51D*)

Living affected individual (proband) with breast or ovarian cancer where the individual + / − family history meets one of the criteria. The proband has:
1 a. Breast cancer (age < 40 years), OR
b. Bilateral breast cancer (age < 50 years), OR
c. Triple negative breast cancer (age < 60 years), OR
d. Male breast cancer (any age), OR
e. Breast cancer (age < 45 years) and a first degree relative with breast cancer (age < 45 years), OR
f. Pathology-adjusted Manchester score ≥ 15 or CanRisk score ≥ 10%
g. Ashkenazi Jewish ancestry and breast cancer at any age
2. Living affected individual with pancreatic cancer AND family history of breast/high grade ovarian/prostate cancer with a pathology adjusted Manchester score of ≥ 15/CanRisk score of 10%
3. Living affected individual with prostate cancer AND a family history of breast/ovarian/pancreatic
cancer with a pathology adjusted Manchester score of ≥ 15/CanRisk score of 10%
4. Deceased affected individual with breast* or high grade ovarian cancer with:
a. A stored DNA, blood or tissue sample available for DNA extraction, AND
b. Pathology-adjusted Manchester score ≥ 17 or CanRisk score ≥ 15%, AND
c. No living affected individual is available for genetic testing
5. Living unaffected individual with:
a. first degree relative affected by breast* or serous ovarian cancer, AND
b. Combined pathology-adjusted Manchester score ≥ 20 or BOADICEA/CanRisk score of ≥ 20% for
affected relative or BOADICEA/CanRisk score of ≥ 10% for unaffected relative AND
c. No living affected individual is available for genetic testing, AND
d. No deceased affected individual with tumour material available for testing
Note for living unaffected individuals:
Where more than one family member may be eligible for unaffected testing, the residual probability of a causative pathogenic variant in the family should be considered, taking into account prior normal unaffected tests

For Lynch syndrome the testing strategy was rather different from the start as pre-screening with either micro-satellite instability (MSI) or mismatch repair immunohistochemistry (IHC) was the usual first step on tumour samples. The original Amsterdam Criteria [11] were developed initially to provide a consistent diagnosis of Lynch Syndrome (previously ‘HNPCC’,-hereditary nonpolyposis colorectal cancer)’. Once germline genetic testing became more available in the late 1990s, the criteria were used to guide testing toward families most likely to harbour a germline variant in one of four mismatch repair genes (MMR) genes. Whilst being fairly specific (about 60% of families had PVs), these criteria lacked sensitivity. Amsterdam criteria were amended in 1999 [12] with extra-colonic cancers added to the diagnostic criteria and looser Bethesda criteria [13] were used to allow inclusion of both tumour testing strategies through MSI/IHC as a pre-screen. This enabled more families to benefit from diagnostic CPG testing [12, 13]. In Manchester we have always tested the germline directly for Lynch syndrome in Amsterdam positive cases as both IHC and MSI miss sufficient numbers to leave a detection rate of 5–10% [14]. In February 2017 NICE recommended pre-screening all colorectal cancers with either MSI or IHC with additional testing for *MLH1* methylation and *BRAF* V600E to rule out somatic involvement of *MLH1*.[15] This was extended to all endometrial cancers following identification of a similar 3% germline mutation rate for MMR genes in the UK to colorectal cancer [16, 17].

For both Lynch and *BRCA1/2* somatic testing of tissue from deceased relatives is a useful mechanism for identification of the family pathogenic variant and is more informative than testing an unaffected at-risk individual [18]. This mechanism is now approved by NHS England [5].

The Manchester Centre for Genomic Medicine (MCGM) serves a population of 5 million in the North-West of England. Outcomes of clinical CPG testing in the clinic, both diagnostic and predictive, have been recorded by the MCGM through the familial registers since diagnostic CPG testing first commenced in 1990 [19, 20]. This record provides a clinical tool to ensure appropriate screening is in place, at risk family members are offered pre-symptomatic CpG testing, as appropriate, and provides a resource for identifying patients when new management approaches or research opportunities become available.

We sought to evaluate, for *BRCA1/2* and MMR genes, time trends of variant detection in index cases, and numbers of cascade tests undertaken in relation to guidance changes.

## Methods

Genetic family registers were interrogated for numbers of diagnostic index tests and subsequent family cascade tests (positive and negative), and year of testing for *BRCA1*, *BRCA2*, *PALB2*, *MLH1*, *MSH2*, *MSH6*, and *PMS2*. Only class 4 (likely pathogenic) and class 5 (pathogenic) CPG variants are presented and are referred to, collectively, as ‘variants’ [21, 22]. Testing of moderate risk genes such as *CHEK2* and *ATM* was never comprehensive until April 2022 and was excluded apart from a small sub-study of CHEK2 variants identified through the family history clinic at Wythenshawe hospital. Data was censored at 6th May 2023. Of note there was much reduced capacity for both laboratory testing and clinic appointments for much of 2020 in view of the SARS-CoV2 pandemic. Uptake of risk reducing surgery (prior to a cancer diagnosis) including Bilateral Risk reducing mastectomy (BRRM) and Risk reducing bilateral salpingo-oophorectomy (RRBSO) was also assessed in those who underwent pre-symptomatic testing (had not had a relevant cancer prior to testing). As all first- degree relatives (FDRs) of a PV heterozygote are recorded on our databases [19] we also assessed overall uptake amongst unaffected FDRs of pre-symptomatic tests. Confirmatory tests in those with a relevant cancer were also recorded.

### Timeline of testing and strategies

The introduction of diagnostic testing for the relevant CPGs in the MCGM began in the early 1995 mirroring the early days of CPG identification when testing was initially undertaken on a research basis prior to formal incorporation into the clinical diagnostic setting in 1998. Thus, testing began for *BRCA1* in 1995 and *BRCA2*, *MLH1* and *MSH2* in 1996.

Initial variant detection strategies were by SSCP, dHPLC and Southern Blot analyses, prior to the introduction of Sanger sequencing in 2003, MLPA for copy number detection in 2005/2006 and next-generation sequencing (NGS) in 2014 (2013 for *BRCA1/2*). A genetic register for *BRCA1/2* and Lynch syndrome was introduced in Manchester in 1996 allowing outreach to relatives to encourage cascade testing. Although only previously referred relatives are directly contacted regarding the availability of testing, index cases are provided with information letters to pass the eligible blood relatives.

## Results

### BRCA1/2 testing

A total of 1600 index *BRCA1/2* variant carriers have been identified (*BRCA1* = 798; *BRCA2* = 802; Table 2). Breast cancer cases predominated but after the 10% NICE threshold meant all non-mucinous epithelial ovarian cancers aged < 60 were eligible in May 2013 the proportion of those tested with ovarian cancer only doubled from 16.5 to 32%. The proportion of pre-symptomatic cases rose from 3.2% before the 2013 NICE guidelines to over 8%. There was also a slight increase in proportion of male index cases rising from 3.5 to > 8% in the last era. Since 2016 30 pathogenic variants have been found from somatic testing of deceased index cases, all but three in ovarian cancer cases.

**Table 2:** Number of positive index cases and additional family tests generate for *BRCA1/2* combined

Era	< 05/2013	05/2013–04/2021	> 04/2021	Total
Number of index cases	782	683	135	1600
Breast cancer index	623	397	89	1109
Ovarian cancer only index	129	223	35	387
% Ovarian only	16.50%	32.65%	25.93%	24.19%
Pre-symptomatic index	25	60	11	96
Obligate carrier index unaffected	5	3	0	8
% Pre-symptomatic	3.20%	8.78%	8.15%	6.00%
Male index	27	34	11	72
% male	3.45%	4.98%	8.15%	4.50%
No tests generated	101	186	84	371
%	12.92%	27.23%	62.22%	23.19%
1 test	155	157	30	342
%	19.82%	22.99%	22.22%	21.38%
2 tests	142	133	14	289
%	18.16%	19.47%	10.37%	18.06%
3–4 tests	183	119	6	308
%	23.40%	17.42%	4.44%	19.25%
5–6 tests	91	55	1	147
%	11.64%	8.05%	0.74%	9.19%
7–9 tests	68	21	0	89
%	8.70%	3.07%	0.00%	5.56%
10 or more	42	12	0	54
%	5.37%	1.76%	0.00%	3.38%
Tests within 2 years	1359	1182	77	2618
Number per family mean	1.74	1.73	0.57	1.64
Total tests generated	2653	1428	77	4158
Mean per family to 9 years	3.39	2.09	0.57	2.60
Tests after 9 years	511	N/a	N/a	
Mean per family	0.65			

### Subsequent non-index cascade tests

We made the main era definition around the NICE guidance of 2013 as this reduced the testing threshold to 10%. In order to allow time for subsequent family testing we separated off those with index positive results after April 2021. The mean number of tests generated was 3.39 for era 1 (< May 2013), 2.09 for era 2 (May 2013–March 2021) and 0.57 for the current era 3. However, when confining tests to within 2 years of family variant report there was a very similar mean of 1.74 for era 1 and 1.73 for era 2 with much of era 3 still not reaching the 2-year follow up. Importantly for era 1 there were 511 additional family tests more than 9 years after index case identification. For number of tests, era 1 had 26% of families generating 5 or more additional tests compared to only 13% in era 2 perhaps reflecting the larger family size of some of the initial families requiring a higher testing threshold.

There were 3568 pre-symptomatic tests and 581 confirmatory tests on people with relevant cancers. Confirmatory tests were far more likely to be carried out within 1 year of the family report 67% versus 32% of all tests in each group (Table 3; *P* < 0.0001). Male versus female ratios were virtually identical. Around one positive additional family test was generated with around 50% of family tests also being positive (Table 4). A total of 556 RRBSO and 453 BRRM were generated in positive female relatives. Overall, of 6170 FDRs eligible for pre-symptomatic testing 2999 (48.6%) underwent testing. This included 940/2899 (32.4%) males and 2059/3171(64.9%) female FDRs (*p* < 0.0001).

**Table 3:** Types of additional tests carried out in families with a positive *BRCA1/2* index case

	Pre-symptomatic	Confirmatory
All	3468	581
Within 1 year	1301	394
% Within 1 year	37.51%	67.81%
1–2 yrs	764	62
> 2yrs	1403	125
Female total	2476	548
Within 1 year	976	372
% Within 1 year	39.42%	67.88%
1–2 yrs	515	58
> 2yrs	985	118
Male total	994	33
Within 1 year	336	22
% Within 1 year	33.80%	66.67%
1–2 yrs	240	4
> 2yrs	418	7
Obligate carrier conformation		109
Female total		65
Male total		44

**Table 4:** Number of positive family tests and subsequent and uptake of risk reducing surgery in non-index cases

Era	< 05/2013	05/2013–04/2021	> 04/2021	Total
Number of index cases	782	683	135	1600
Number of positive female non-index positive tests	1032	484	34	1550
Mean per index	1.32	0.71	0.25	0.97
Number of RRBSO	455	100	1	556
% of positive	44.09%	14.64%	0.74%	34.75%
Number of BRRM	361	92	0	453
%	34.98%	13.47%	0.00%	28.31%
Number of breast cancer diagnosis post pre-symptomatic testing	78	31	1	110
Number undergoing contralateral mastectomy	51 (65.4%)	13 (42%)	0	64 (58.2%)
Number of female negative	1017	498	22	1537
% positive	50.37%	49.29%	60.71%	50.21%
Number of male positives	318	250	14	582
Number of male negatives	286	196	7	489
% positive	52.65%	56.05%	66.67%	54.34%

### Lynch syndrome

A total of 625 index *MMR* variant carriers have been identified (*MLH11* = 185; *MSH2* = 238; *MSH6* = *132; PMS2* = *70;* Table 5). Colorectal cancer cases predominated but from 2013 a higher proportion of endometrial index cases were identified with close to ¼ since April 2021 when the NICE endometrial guideline for Lynch testing was instituted. As expected with Lynch affecting men almost the same as women nearly 50% of index cases were male. Since 2016, 16 pathogenic variants have been found from somatic testing of deceased index cases mostly in colorectal cancer. In all these 625 index cases have led to the identification of 857 family members positive for the MMR PGV.

**Table 5:** Number of positive index cases and additional family tests generate for Lynch MMR genes combined

era	< 05/2013	05/2013–04/2021	> 04/2021	Total
Number of index cases	306	262	57	625
Colorectal cancer	240	171	35	446
Endometrial cancer only	30	36	14	80
% Endometrial only	9.80%	13.74%	24.56%	12.80%
Other cancer index	17	37	8	62
Pre-symptomatic	19	18	0	37
Obligate carrier	0	0	0	0
% pre-symptomatic	6.21%	6.87%	0.00%	5.92%
Male index	142	121	27	290
% male	46.41%	46.18%	47.37%	46.40%
No tests generated	32	41	34	107
%	10.46%	15.65%	25.19%	17.12%
1 test	67	75	10	152
%	21.90%	28.63%	7.41%	24.32%
2 tests	49	49	8	106
%	16.01%	18.70%	5.93%	16.96%
3–4 tests	80	52	3	135
%	26.14%	19.85%	2.22%	21.60%
5–6 tests	37	28	2	67
%	12.09%	10.69%	1.48%	10.72%
7–9 tests	28	10	0	38
%	9.15%	3.82%	0.00%	6.08%
10 or more	13	7	0	20
%	4.25%	2.67%	0.00%	3.20%
tests within 2 years	498	557	46	1101
Number per family mean	1.63	2.13	0.81	1.76
Total tests generated within 9 years	1054	668	46	1768
Mean per family to 9 years	3.44	2.55	0.81	2.83
Tests after 9 years	216	N/a	N/a	

### Subsequent non-index cascade tests

To compare with HBOC we used the same 2013 cut off. In order to allow time for subsequent family testing we separated off those with index positive results after April 2021. The mean number of tests generated was 3.44 for era 1 (< May 2013), 2.55 for era 2 (May 2013–March 2021) and 0.81 for the current era 3. This was essentially the same as for HBOC in era 1 but with more tests per index being generated after for Lynch. When confining tests to within 2 years of family variant report there was a very similar mean of 1.63 compared to HBOC was seen for era 1 but this increased to 2.13 compared to 1.73 for HBOC in era 2 with much of era 3 still not reaching the 2-year follow up. Importantly for era 1 there were 216 additional family tests more than 9 years after index case identification. For number of tests, era 1 had 25.5% of families generating 5 or more additional tests virtually identical to HBOC compared to only 17.2 a little higher than the 13% in era 2 for HBOC perhaps again reflecting the larger family size of some of the initial families requiring a higher testing threshold.

There were 1514 pre-symptomatic tests and 249 confirmatory tests on people with relevant cancers. Confirmatory tests were again far more likely to be carried out than pre-symptomatic within 1 year of the family report 147/249 (59% Vs 602/1514 (39.8%) (*P* < 0.0001), though the difference was less marked than for HBOC. The proportion of male pre-symptomatic tests was also much higher than for *BRCA1/2* at with 639/1514 (42%) male for Lynch compared to 1058/4158 (25.8%) for *BRCA1/2* (*P* < 0.0001)*.* For FDRs uptake of pre-symptomatic tests was 63.4% in males (568/896) compared to 75% (766/1022) *p* < 0.0001. The uptake in male FDRs was much higher than in *BRCA1/2* (*p* < 0.0001), but the 75% uptake in females was still significantly higher than the 64.9% for *BRCA1/2* (*p* < 0.0001),

### *CHEK2* as a moderate risk gene

Although *CHEK2* testing outside the index case has not been systematically offered some testing through the Manchester family history clinic has occurred through the FHrisk study. In total 37 index cases have been identified, but only 36 additional family members were tested clinically until very recent testing of stored blood DNA samples from a further 13 women affected with breast cancer. In total of affected non-index cases only 50% 11/22 tested positive for the *CHEK2* variant. This compares with 252/290 (87%) for *BRCA1* (*p* < 0.0001) and 328/390 (84%) for *BRCA2* (*p* = 0.0004).

### Proportion of index cases found on mainstream testing

Mainstream testing in oncology and surgery started in November 2017 with the focus on driving treatment decisions for the index case rather than cascading. The proportion of index cases found by cascading has increased for *BRCA1/2* from 2% in 2017 to 53% in 2019 and 84% so far in 2023 (supplementary Table 1).

## Discussion

The provision of CPG testing has evolved enormously revolutionising the practice of clinical cancer genetics over the past 30 years. Where an index case CPG variant is found this enables (i) cancer prevention and early detection strategies for family members at-risk as well as for second cancers in the index case (ii) management strategies in the index case, including surgical management and targeted therapeutic strategies [23]. The focus has very much switched from emphasis on cascade testing for relatives to management of the index case since 2017. This is reflected in the high proportion of index cases found in 2023 from mainstream testing. Although, cascading still occurs from referral of the index case to genetics, this may be slower or may not occur due to the index case being too unwell or refusing referral reducing the rate of family testing [6]. We still do not have enough data to determine whether long term uptake of testing following mainstream testing is lower than the traditional genetics referral model. In era 1 the main motivation for testing was to inform family members of their risk (often generated by unaffected people attending concerned about their risks). Targeted therapies including PARP inhibitors for *BRCA1/2* deficient ovarian and more recently breast cancers and PD-1 inhibitors for MMR deficient tumours, were not available until towards the end of era 2 or even era 3 [24, 25]. Changes due to mainstreaming has also changed the cancers from which index cases have suffered, with ovarian cancer becoming more frequent in *BRCA1/2* and endometrial cancer in Lynch syndrome.

Decreases in family size from reducing the thresholds for testing from 20% through 10% to effectively only 3% for women with an isolated non-mucinous ovarian cancer over age 70 [26], has meant fewer at-risk relatives per family available for cascading. This is reflected in the larger proportion of families with high numbers of generated cascade pre-symptomatic testing in the earlier era (< 2013) with 14.1% of families generating ≥ 7 tests compared to only 4.8% of families in era 2. Although there were obviously fewer years to generate these tests there were still a higher mean number per family tested to the 9-year point in era 1 compared to era 2 2013–0/4/2021). Nonetheless there were still a similar number of tests generated per family in the first two years at 1.74 and 1.73 respectively. A similar number of pre-symptomatic tests were generated per family for the MMR genes both at 2 years and overall.

The situation in MCGM prior to therapeutic options being available is reflective of the whole UK and is similar internationally. Therefore, consensus strategies were put in place to identify those individuals and families in whom there was the greatest likelihood of detecting a causative germline variant and who would most likely benefit from genetic investigations. This is reflected in NICE guidelines for breast cancer [4] and the use of the original Amsterdam Criteria [11] to drive testing for MMR genes.

CPG variants of the *BRCA1*, *BRCA2* and MMR CPGs are by far the most common of all CPG variants identified in testing with combined population frequencies of 0.3–0.5% for *BRCA1/2* and Lynch [27, 28]. The lower number of identified CPG index cases identified for other genes reflects the rarity of these variants for example STK11, CDH1 and PTEN, but also the reduced actionability of the moderate risk genes. Until recently cascading was not offered for either *ATM* or *CHEK2* in the UK. Indeed, although their combined frequency exceeds *BRCA1/2* [28] they were only included officially on NHS England breast cancer gene panels in 2021 [5]. The main issue was how to counsel family members on their risk if they test positive or negative. This is reflected in the low proportion of confirmatory tests that were positive (50%) for *CHEK2* compared to over 80% for *BRCA1, BRCA2* and *PALB2* [9]. The availability of breast cancer risk estimates using the CanRisk programme [10] is likely to increase uptake of cascading as cancer genetics professionals now feel more confident providing this service.

As expected, we found higher uptake in males for Lynch syndrome than for *BRCA1/2* [27] with a higher proportion of pre-symptomatic tests in males at 42% of all tests in males for Lynch compared to 25.8% for *BRCA1/2* (*P* < 0.0001). Overall uptake was also higher in males FDRs. This higher uptake likely reflects the increased utility of testing in males for personal cancer risks particularly for colonoscopy screening. This is consistent with our previous publications on uptake in both conditions [29, 30]. There were also a number of tests that occurred more than 9 years post index test. This can reflect ineligibility due to being < 18 years at date of index case but also a slower initial uptake between 18 and 25 years of age [29].

Here we have presented the cascading of CPG testing for 33 years at MCGM in the UK. With the rapid increase in mainstreaming of diagnostic testing where immediate treatment decisions are required, pathways need to ensure at-risk family members are offered testing. It is notable that the detection of 2225 index cases for *BRCA1/2* and Lynch syndrome combined at MCGM has led to the subsequent identification of over 2989 positive family members, who can then benefit from cancer prevention and early detection strategies resulting in both individual, and societal health economic benefit, Equally important is the reassurance for close to 3000 family members testing negative as well as all their offspring who, for the most part can be reassured regarding risk.

### Supplementary Information

Below is the link to the electronic supplementary material.

Supplementary file1 (DOCX 12 kb)
