# Supplementary material for: Cascade screening in HBOC and Lynch syndrome: guidelines and procedures in a UK centre
Source: Fam Cancer. 2024 Mar 13;23(2):187–95. doi: 10.1007/s10689-024-00360-9 (PMC11153258; doi:10.1007/s10689-024-00360-9)
Supplement: Supplementary file 1 — Supplementary file1 (DOCX 12 kb) [file 10689_2024_360_MOESM1_ESM.docx]

**Supplementary table 1:** Proportion of *BRCA1/2* index identified from mainstream testing.

| year | number *BRCA1/2* index identified | mainstream | genetics | Mainstream % |
| --- | --- | --- | --- | --- |
| 2016 | 50 | 0 | 50 | 0.0% |
| 2017 | 53 | 1 | 52 | 1.9% |
| 2018 | 81 | 14 | 67 | 17.3% |
| 2019 | 74 | 39 | 35 | 52.7% |
| 2020 | 50 | 21 | 29 | 42.0% |
| 2021 | 69 | 42 | 27 | 60.9% |
| 2022 | 75 | 52 | 23 | 69.3% |
| 2023 | 69 | 58 | 11 | 84.1% |
